# The contribution of twin studies to the understanding of the aetiology of asthma and atopic diseases

**DOI:** 10.3402/ecrj.v2.27803

**Published:** 2015-09-11

**Authors:** Simon F. Thomsen

**Affiliations:** Department of Respiratory Medicine, Bispebjerg Hospital, Copenhagen, Denmark

**Keywords:** Asthma, atopic dermatitis, atopic diseases, environment, epidemiology, genetic, hay fever, twin study

## Abstract

The prevalence of asthma and other atopic diseases has increased markedly during the past decades and the reasons for this are not fully understood. Asthma is still increasing in many parts of the world, notably in developing countries, and this emphasizes the importance of continuing research aimed at studying the aetiological factors of the disease and the causes of its increase in prevalence. Twin studies enable investigations into the genetic and environmental causes of individual variation in multifactorial diseases such as asthma. Thorough insight into these causes is important as this will ultimately guide the development of preventive strategies and targeted therapies. This review explores the contribution of twin studies to the understanding of the aetiology of asthma and atopic diseases.

The prevalence of asthma has increased markedly during the past decades and the reasons for this are not fully understood. Asthma is still increasing in many parts of the world, notably in developing countries and this emphasizes the importance of continuing research aimed at studying the aetiological factors of the disease and the causes of its increase in prevalence (1).

Twin studies enable investigations into the genetic and environmental causes of variation in multifactorial diseases such as asthma. The premise of the twin method is that monozygotic (MZ) twins not only share all their genes but also their upbringing and early environment. Conversely, apart from their upbringing and early environment, dizygotic (DZ) twins share an average of only 50% of their segregating genes. Therefore, all phenotypic dissimilarity between MZ twins is assumed to be due to non-shared environmental differences between the twins, whereas dissimilarity between DZ twins is assumed to be due both to genetic and non-shared environmental differences. Consequently, if MZ twins are more *similar* for asthma than DZ twins, a genetic contribution to the trait can be inferred. However, a caveat to the interpretation of twin studies is the phenomenon of epigenetics; the transcriptional dynamic alterations leading to changes in gene expression, observed as phenotypic dissimilarities not only in DZ twins but also in MZ twins.

Understanding of the aetiology of asthma is important as this will ultimately guide the development of preventive strategies and targeted therapies. This review explores the contribution of twin studies to the understanding of the aetiology of asthma with special emphasis on the importance of genetic and environmental factors for the variation in the susceptibility to asthma; on how twin studies have corroborated theories explaining asthma's recent increase in prevalence; and on how these fit with the explanations of the epidemiological trends in other common chronic diseases of modernity. For a more comprehensive review of this topic, the reader is referred to a previous publication (2).

## Genetic and environmental influence on asthma

**Fig. 1:**
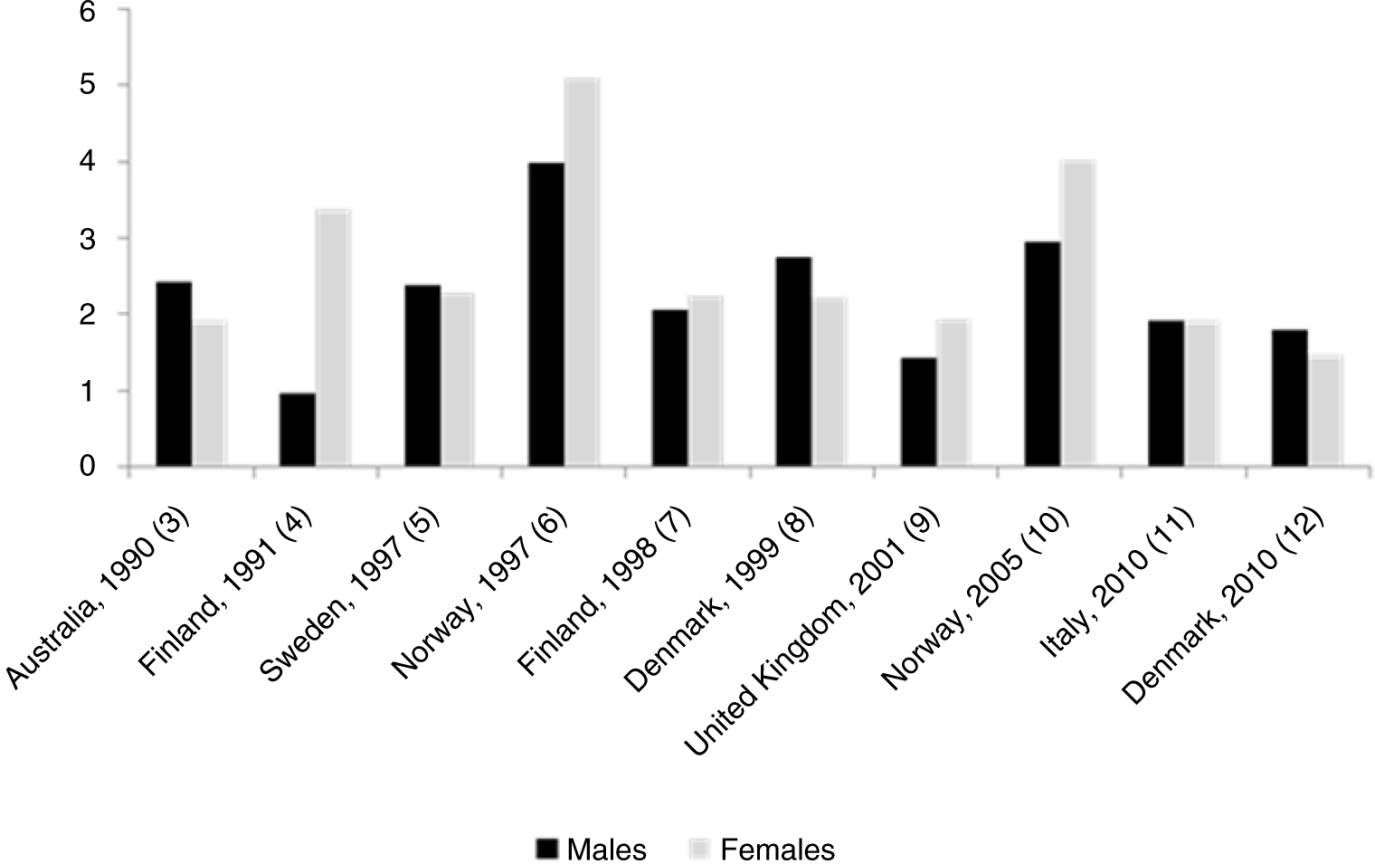
Ratio between MZ and DZ concordance rates for asthma obtained from recent population-based twin studies.

Several twin studies have examined the heritability of asthma. For MZ twins, concordance rates range is 0.08–0.66, while for DZ twins the range is 0.05–0.45. However, as the concordance depends on the prevalence of the disease, and since the studies represent a period in which asthma has increased markedly in prevalence, the *ratio* between MZ and DZ concordances signals more accurately the genetic influence on asthma (Fig. 1) (3–12). Most previous studies show that MZ twins are more concordant for asthma than are DZ twins with a ratio between these concordances of around two (12). One small twin study combining data from the United States and Finland found similar concordance rates for asthma in MZ twins *reared apart* compared with MZ twins *reared together*, suggesting that shared environment has very little effect on the development of asthma (13). Despite heterogeneity between different twin studies regarding diagnostic criteria of asthma, age and year of examination, and country of origin, the collective evidence is consistent with asthma being a highly heritable disease with genetic factors accounting for approximately 60–80% of its susceptibility and with only a modest or no effect attributable to environmental effects shared between family members. However, an important caveat of twin studies is that a high heritability does not preclude an important contribution of environmental factors to asthma.

A few studies have examined more elaborated aspects of the phenotypic expression of asthma. For example among Danish twins, the correlation between the ages at onset of asthma was higher in MZ twins than that in DZ twins, and with a heritability of age at onset of asthma of 35% (14). This finding indicates that the degree of genetic relatedness between family members dictates the expected waiting time to onset of asthma in the second family member after onset of asthma in the first. Moreover, the stronger a person's genetic predisposition for asthma, the earlier he or she developed the disease, that is, if the first member of a twin pair develops asthma at an early age, then the co-twin also has a higher risk of developing asthma at an early age, whereas late-onset asthma was less dependent on asthma status of the co-twin (14). Another study of Danish adult twins estimated the genetic and environmental influence on asthma symptomatology using clinical data (15). In this study, the overall symptomatic severity of asthma was correlated more among MZ twin pairs than among DZ twin pairs concordant for asthma. Individual asthma symptoms, particularly wheezing, shortness of breath, and cough were also correlated to a higher extent among MZ than among DZ twin pairs concordant for asthma. It was found that genetic factors explained 24% of the variation in overall asthma symptom severity, whereas non-shared environment accounted for the remaining 76%, indicating that random variation and variation due to specific environmental exposures account for the main part of the variation in asthma symptom severity between individuals.

The same study of Danish twins (15) also found considerable heritability for several intermediate phenotypes associated with asthma; such as lung function, airway responsiveness, and exhaled nitric oxide (Table 1). Collectively, the results from the Danish and other twin studies imply that genetic factors contribute to the stability of pulmonary function over time, whereas environmental factors contribute to its change (i.e. reduction). One such factor may be smoking, which has been shown to modify the genetic influence on FEV_1_ in adult twins (18).

**Table 1:** Heritability of intermediate asthma phenotypes in adult twins

	Heritability (%)
FEV_1_	68*
FVC	58*
FEV_1_/FVC	22
DRS_methacholine_	43*
FeNO	67*
Serum total IgE	81*
Positive SPT	54*
HDM sensitization	6
Serum eosinophil cationic protein	57*
Serum tryptase	82*

Serum tryptase Ref. (16); serum eosinophil cationic protein Ref. (17).

* *p*<0.001.

Another important question addressed in twin studies is whether the inherited tendency to become allergic (to be atopic) extends to specific allergens. Several twin studies of recent and older dates have shown that the risk of atopy as well as the variation in serum total IgE has moderate-to-high heritability (19, 20). Interestingly, a study of 57 Danish twins showed that as much as 80% of the variation in cord blood IgE was ascribable to genetic effects, indicating a substantial influence of genetic factors for this trait already before the onset of clinical allergic disease (21). However, follow-up of this cohort showed that the correlation between cord blood IgE and serum total IgE at age 6–9 years was close to zero, indicating that different effector mechanisms may be operating at different ages (22). Taken together, twin studies provide strong evidence for genetic determination of atopy but limited evidence to support inheritance of specific allergies such as, for example, house dust mite and grass allergy.

## The relationship between the atopic diseases

The sequential development of the atopic diseases, that is, atopic dermatitis, asthma, and allergic rhinitis, is referred to as the *atopic march*, characterised by the progression of atopic dermatitis to asthma and allergic rhinitis during the first years of life (23). While this trajectory is well documented, controversy remains as to whether the atopic diseases are causally related or whether they are diverse clinical manifestations of a common underlying, genetic, disease trait.

Recent discoveries have led to the formulation of a *leaky barrier hypothesis* stating that the skin acts as the site of primary sensitization through defects in the epidermal barrier with secondary reactivity in the airways (23). Compiling evidence centres on inherited defects in the skin barrier protein *filaggrin* as initiater of the atopic march (24). Filaggrin is expressed in the skin, the oral mucosa, and the nasal vestibule, but apparently *not* in the bronchial or the gastrointestinal epithelium (25). Therefore, loss-of-function mutations in the filaggrin gene (*FLG*) are unlikely to directly affect barrier function and allergen reactivity in the lungs or other distant target organs. Instead, filaggrin-deficiency driven primary percutaneous allergic sensitization is speculated to lead secondarily to hyperactive airways and asthma (24, 25).

*FLG* mutations are present in ~10% of Europeans but in as many as half of all patients with atopic dermatitis (26). Notably, the risk of atopic dermatitis among individuals with *FLG* defects is increased about two times in family studies and almost five times in case-control studies (27). Moreover, the risk of allergic sensitization, allergic rhinitis, and asthma is also increased but *only* in those with coexistent atopic dermatitis. These findings support that, at least in a subset of those with atopic disease, *FLG* defects may be the fundamental predisposing factor not only for the development of eczema but also for initial sensitization and progression of allergic disease (27).

Twin studies have shown that most of the association between the atopic diseases can be explained by a shared genetic liability. This is exemplified by the observation that MZ twins are often more concordant for different pairs of atopic diseases (e.g. atopic dermatitis and asthma) than are DZ twins. Specifically, in a large sample of Danish twins 12–41 years of age, as much as 81% of the phenotypic relationship between atopic dermatitis and asthma was mediated through pleiotropic genetic effects, whereas 85% of the relationship between atopic dermatitis and hay fever, and 70% of the relationship between asthma and hay fever, was ascribable to such common genetic effects (28). These findings are consistent across age groups and countries (3, 5, 11, 29, 30). A direct interpretation of this is that the susceptibility to the different atopic diseases is largely determined by a common set of genetic factors and to a lesser extent also by disease-specific or disease-modulating genetic factors.

This lends support to the hypothesis of a common genetic atopic disease trait, mediated through mutations in skin barrier proteins, of which atopic dermatitis, asthma, and allergic rhinitis, respectively, are causally independent but sequentially occurring manifestations. However, the chain of events that links asthma to atopic dermatitis and allergic rhinitis is complex and involves a multitude of hereditary and developmental factors that exert their effect in the context of environmental exposures. However, this course may be relevant only for certain types of asthma, particularly classical atopic asthma with early onset, whereas adult-onset asthma or non-atopic asthma may result from different pathways.

## The hygiene hypothesis

During the second half of the last century, the occurrence of asthma and other atopic diseases increased considerably worldwide. Changes in lifestyle and environment, so-called *Westernisation*, have been postulated as the primary cause for this, mainly since the rising incidence of atopic diseases has occurred more rapidly than changes to the genome sequence would allow (31, 32).

Clues to the causes of this widespread increase in asthma prevalence come from studies of migrants. More specifically, immigrants to the industrialized world from the developing world increasingly develop allergic disorders in relation to the length of time since arrival in the industrialized world (33, 34). For example, the prevalence of asthma was significantly higher in West Germany compared with East Germany shortly after their reunification, suggesting an impact of differential environments on two ethnically similar populations (35). Only a few years later, prevalence rates had converged as an indication that more congruent lifestyles had developed (36). Also, the frequency of allergic diseases is different in Finnish and Russian Karelia, two neighbouring geographical regions with the same ethnic background: Finland has a fivefold higher allergy incidence (37). These results suggest an environmental change-induced increase of gene expression in the studied populations leading to an increased occurrence of asthma over time (38, 39).

A Danish twin study has provided evidence in favour of this hypothesis being the first to study changes in prevalence and heritability of asthma over time (40). In this study of Danish adolescent twins, the prevalence of self-reported asthma increased from 7.1 to 10.8% between 1994 and 2003. In the same period, the heritability of asthma increased significantly from 79 to 91%. This was particularly due to an increased concordance for asthma among MZ twins in 2003 compared with 1994 (0.73 vs. 0.50), whereas the concordance for asthma among DZ twins was more or less unchanged between 2003 and 1994 (0.29 vs. 0.24); the ratio between concordance rates in MZ and DZ twins increased from 2.08 to 2.52 during these years. These results fit well with the hypothesis that the prevalence of asthma has increased globally due to widespread environmental changes. Notably, the influence of genetic factors seems to have increased over time as a result of environmental changes. That is, the extent to which genetic influences affect asthma has increased as a reaction to these environmental changes leading to a higher heritability of asthma in the more recent generations (40).

Intriguingly, the *hygiene hypothesis* (41) has been extended to include inflammatory diseases in general, particularly several *T*_*H*_ *1-mediated* autoimmune diseases, such as multiple sclerosis, inflammatory bowel disease and type 1 diabetes (42). These diseases show remarkably similar geographical distributions and epidemiological patterns compared with the atopic diseases (32, 42). A low prevalence of autoimmune diseases is chiefly observed in the tropical regions, where infections are prominent, whereas in more temperate regions their occurrence is high (43). Interestingly, as atopic diseases and autoimmune diseases seem to follow similar epidemiological trends, they are expected to be inversely related on the individual level. Notably, while atopic diseases are dominated by production of T_H_2 cytokines, such as IL-4, IL-5, and IL-13, autoimmune diseases, such as type 1 diabetes, are dominated by the T_H_1 cytokines IL-2 and interferon gamma (IFN-γ). However, this T_H_1/T_H_2 dichotomy represents a simplified view of the immunological mechanisms underlying these diseases. For example, in *chronic* asthma – unlike in acute asthma – T_H_1 cytokines have also been shown to play a prominent role (44), whereas in type 1 diabetes, T_H_2 mechanisms are important (45). Furthermore, other immune cells, such as regulatory T cells and T_H_17 cells and their respective cytokines, as well as aspects of the innate immune system, have similarly been shown to play important roles in the pathogenesis both of asthma (44) and autoimmune diseases, such as type 1 diabetes (46).

An inverse association between atopic diseases and type 1 diabetes has been found in several, albeit not all (47), observational studies of singleton populations, both in relation to asthma, hay fever, and atopic dermatitis and also in relation to allergic sensitization (48). However, only one twin study has examined this (49): in Danish child and adolescent twins with hospital diagnosed type 1 diabetes, the risk of self-reported asthma was found to be slightly, but not statistically significantly, lower compared with twins without type 1 diabetes (9.5% vs. 11.2%), supporting that asthma *in children* may be inversely related to type 1 diabetes. A composite measure of any self-reported atopic disease, that is, asthma, hay fever and/or atopic dermatitis, was significantly less prevalent in child and adolescent twins with type 1 diabetes compared with non-diabetic individuals (11.9% vs. 28.0%). This effect was driven primarily by a strong inverse relationship between atopic dermatitis and type 1 diabetes, which was present both in children and adults; the prevalence of atopic dermatitis in individuals with and without type 1 diabetes was 2.1 and 9.9%, respectively. Of particular note was a significant negative genetic correlation between type 1 diabetes and atopic dermatitis of −0.30 and a substantial positive, albeit not statistically significant, non-shared environmental correlation of 0.52, indicating that atopic dermatitis and type 1 diabetes are regulated partly by opposing genetic mechanisms but, in contrast, seem to share environmental risk factors to a sizable extent (49). This observation seems to fit well with the hygiene hypothesis as a coherent explanation for the recent increase in the prevalence both of atopic diseases and autoimmune diseases that also accommodates the contrasting risk for these diseases within the individual (the T_H_1/T_H_2 paradigm).

Studies of infant bronchiolitis, particularly caused by *respiratory syncytial virus* (RSV), have shown that respiratory viral infections encountered early in life constitute an exception to the hypothetical rule that microbial stimulation confers a protective effect on asthma development. RSV affects most children at some point during their first 2 years of life, and it has been estimated that at least 33.8 million episodes of RSV-associated acute lower respiratory infection occur worldwide per year in children younger than 5 years of age (50).

It is not clear why only a small number of RSV-infected children develop severe respiratory disease requiring hospitalisation, whereas the majority has only mild disease. However, prematurity, T cell immunodeficiency, chronic lung disease, and congenital heart disease are established risk factors (51). Genetic background also determines the clinical outcome of RSV infection. A Danish twin study of 3–9-year-old children found that genetic effects explained approximately 20% of the variation in the risk of RSV hospitalisation (52). In contrast, common environmental effects, highlighting the infectious nature of RSV, explained most of the remainder of the variation.

Severe RSV infection in infancy is a firmly established risk factor for subsequent asthma, wheezing, and abnormal pulmonary function in later childhood (53). Of note, a systematic review and meta-analysis of observational studies comprising ~82,000 individuals, showed that children who had had severe RSV disease in early life have an approximately fourfold higher incidence of asthma in later childhood, but with a decrease in risk with age at follow-up (54). Contrary to these findings, several studies have found that predisposition to asthma increases the risk of lower respiratory tract infection and RSV hospitalisation. For example, early wheezy symptoms were found to be a strong risk factor for subsequent RSV hospitalisation (55), whereas impaired pulmonary function (56) and airway hyperresponsiveness (57) measured at 1 month of age increased the risk of acute severe bronchiolitis in response to infections with respiratory tract viruses, particularly RSV, in later childhood. Finally, several of the genetic variants associated with severe RSV infection have also been implicated in the susceptibility to asthma (53).

While the strength of the association between severe RSV infection and asthma is well described, the nature of this association remains imperfectly understood. It is unclear whether severe infant RSV infection plays a direct causative role in asthma or simply unmasks a genetic predisposition for subsequent asthma, wheezing, and reduced pulmonary function later in life. Several Danish twin studies have addressed this problem (58–60). Particularly, a population-based study of 3–9-year-old twins (8,280 pairs) (58) showed that the association between severe RSV infection and asthma was stronger in MZ than in DZ twin pairs. In fact, it was shown that the association between severe RSV infection and asthma could be ascribed to genetic effects shared between the two disorders. Moreover, modelling the direction of causation between severe RSV infection and asthma showed that a model in which asthma was assumed to ‘cause’ severe RSV infection fitted the data significantly better than did a model in which severe RSV infection was assumed to ‘cause’ asthma. This conclusion is consistent with the hypothesis that severe RSV infection seems to be an indicator of the individual genetic susceptibility to asthma rather than a direct cause of asthma. However, as severe RSV infection does not seem to directly ‘cause’ asthma (as the pathways leading to asthma are multifactorial), modelling for the direction of causation does not necessarily prove a non-relation; it merely implicates an existing but not strong relationship.

Furthermore, in a clinical follow-up study nested within the larger population of Danish twins, 3–9 years of age, 37 MZ twin pairs discordant for RSV hospitalisation at a mean age of 10.6 months were studied for the possible subsequent development of asthma, lung function impairment and atopy at a mean age of 7.6 years (60). There were no differences observed between the RSV-hospitalised MZ twin and the non-hospitalised co-twin in any of the outcomes studied (asthma, wheezing, atopic dermatitis, airway responsiveness, positive SPT, FEV_1_, or FeNO), substantiating that severe RSV infection does not seem to directly cause asthma or other atopy-related conditions.

## Foetal programming of asthma

In 1989, Barker and colleagues observed that low birth weight was associated with death from ischaemic heart disease at adult age (61). This observation gave rise to the *foetal programming (Barker) hypothesis*, the phenomenon whereby malnutrition and other adverse influences in utero permanently set the structure of different organs and the function of different key systems and through these mechanisms predetermine a person's risk of chronic diseases later in life (62). There is a large body of experimental and epidemiological evidence that demonstrates this phenomenon, both in relation to cardiovascular and metabolic diseases, and also in relation to atopic diseases, autoimmune diseases, psychiatric diseases, and several cancers (62).

Interestingly, twins are born an average of 1,000 g lighter than singletons and 3 weeks preterm (63). Moreover, the intrauterine environment of twins differs from that of singletons in a number of ways that, according to the foetal programming hypothesis, could be speculated to put twins at a higher risk of asthma and other chronic diseases later in life. According to this hypothesis, MZ twins would be speculated to have an even higher risk of asthma compared with DZ twins because of a more hostile foetal environment, particularly for the smaller twin.

Among Danish twins, the risk of asthma was slightly, albeit not statistically significantly, higher among MZ twins compared with DZ twins, which would signal that MZ twins experience a more adverse foetal environment than do DZ twins (12). However, seemingly the low birth weight and the adverse intrauterine environment of twins do not result in a higher risk of asthma in twins as a whole compared with singletons. Rather, there may be a physiological downregulation of foetal growth in twins starting in early gestation; and early embryonic development in twins may be timed slightly differently compared with singletons in order to avoid long-term negative effects of intrauterine growth retardation (64). This has been exemplified in a few studies that have compared the prevalence of asthma in twins and singletons using similar methodology. Interestingly, a Swedish study of conscripts found a *reduced* risk of asthma in twins compared with singletons (65), whereas a study from the United Kingdom found a reduced risk of hospitalisation for asthma among small twin children (66). An alternative interpretation of these studies is that the opportunity for cross-infection between twins may lead to an overall lower risk of asthma in twins as a whole. However, other factors, for example socioeconomic differences between twin and singleton families, may also explain these observations (67); moreover, comparison of prevalence estimates in twins and singletons from other countries, ignoring diagnostic differences, seems not to reveal a different risk of asthma in twins in general compared with singletons (68). This is consistent with findings regarding other diseases and overall mortality in twins and singletons (63).

**Fig. 2:**
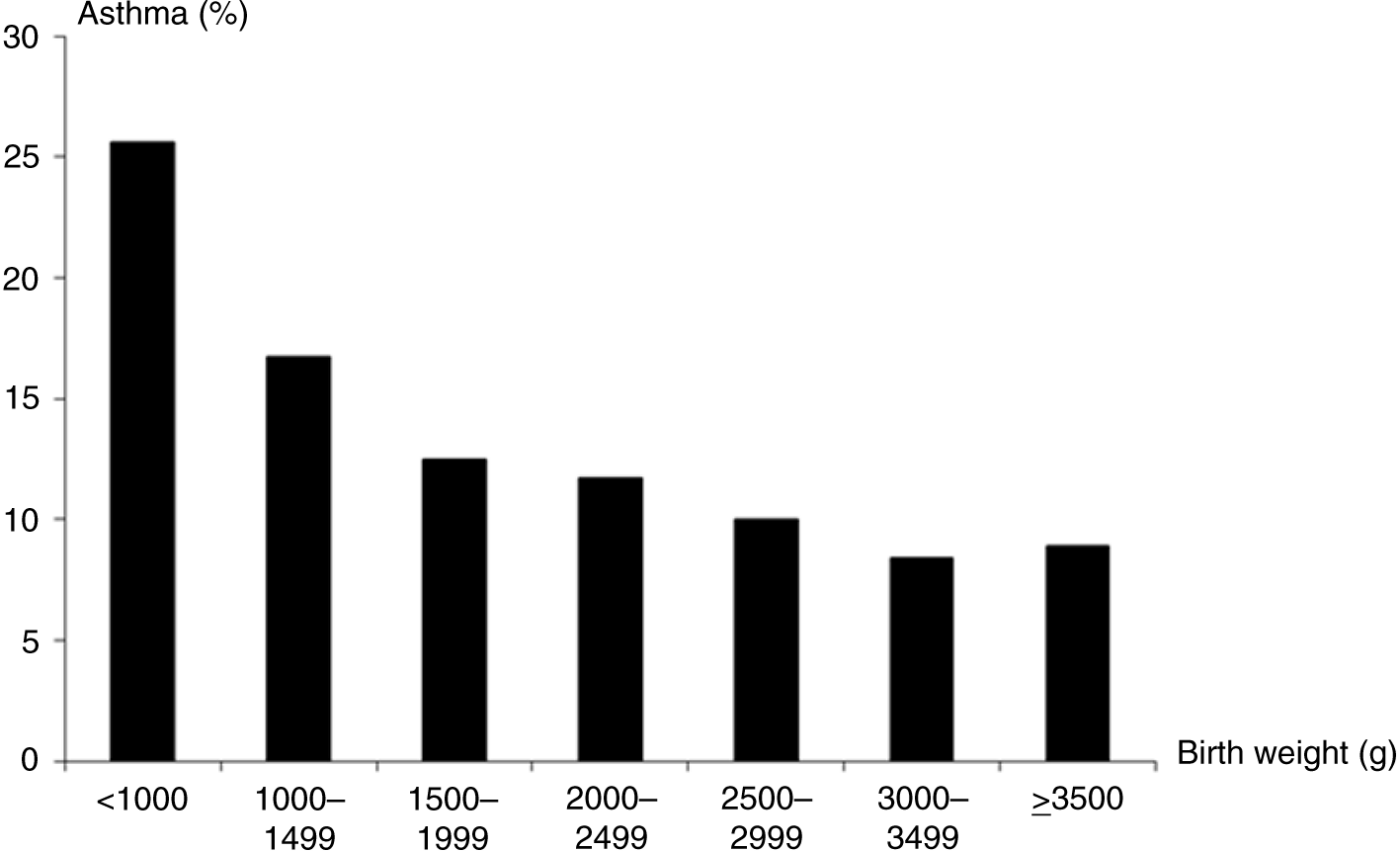
Relationship between asthma and birth weight in Danish twins, 3–9 years of age.

A population-based study of Danish twins (8,280 pairs) showed that children with a history of asthma at age 3–9 years weighed on average 122 g less at birth compared with children who had not developed asthma (69). There was a linear increase in asthma risk with decreasing birth weight; for every 100 g *decrease* in birth weight the risk of asthma *increased* by 4% (Fig. 2). Within twin pairs, the lower birth weight twin had a significantly *increased* risk of asthma compared with the heavier co-twin (11.3% vs. 9.9%) after adjustment for sex, birth length, and Apgar score. Notably, the risk of asthma tended to be *higher* in the lower birth weight MZ than DZ co-twin relative to the higher birth weight twin, especially for large intrapair differences in birth weight, suggesting that the relationship between low birth weight and asthma was mediated by *non-genetic* factors, and *not* by a common genotype underlying the association between low birth weight and asthma. This effect has also been observed among Finnish (70) and Swedish (71, 72) twins, suggesting that low birth weight *per se* is not likely to be the causal factor leading to asthma. Instead, the association between low birth weight and asthma may be explained by early adaptation mechanisms in response to various adverse exposures in foetal life and early childhood.

## Association between asthma and obesity

While the relationship between obesity and cardiovascular disease is well described, recent years have seen the identification of a link between obesity and other noncommunicable diseases, such as asthma, several cancers, gastroesophageal reflux disease, liver disease, neuropsychiatric diseases, psoriasis, and sleep disorders (73). Indeed, asthma in the obese population may be considered a distinct clinical phenotype, characterised by later onset, female preponderance, greater symptomatology, decreased sensitivity to inhaled corticosteroids, and possibly also by a relatively low degree of eosinophilic inflammation compared with classical atopic asthma (74).

There is a consistent but varying strength of association between obesity and asthma across populations, with a dose-dependent risk of asthma according to increasing BMI, even within the normal range of BMI, and with a stronger relationship to self-reported asthma and asthma symptoms compared with intermediate asthma phenotypes such as airway hyperresponsiveness and atopy. A systematic review and meta-analysis of *prospective* population studies found an increased risk of incident asthma in obese individuals, both among men (46% increased risk) and women (68% increased risk) (75). Type 2 diabetes has also been associated with asthma (76, 77) and asthma symptoms (78) in some population studies, but not in all (79), as has insulin resistance (80–82). A recent large population study of ~85,000 adults from Spain showed that elevated waist circumference (or BMI), elevated serum triglyceride and low serum high-density lipoprotein (HDL) were significantly associated with wheezing, and with stronger associations in individuals without concomitant rhinitis symptoms, that is, in those who could be considered non-atopic (83).

**Fig. 3:**
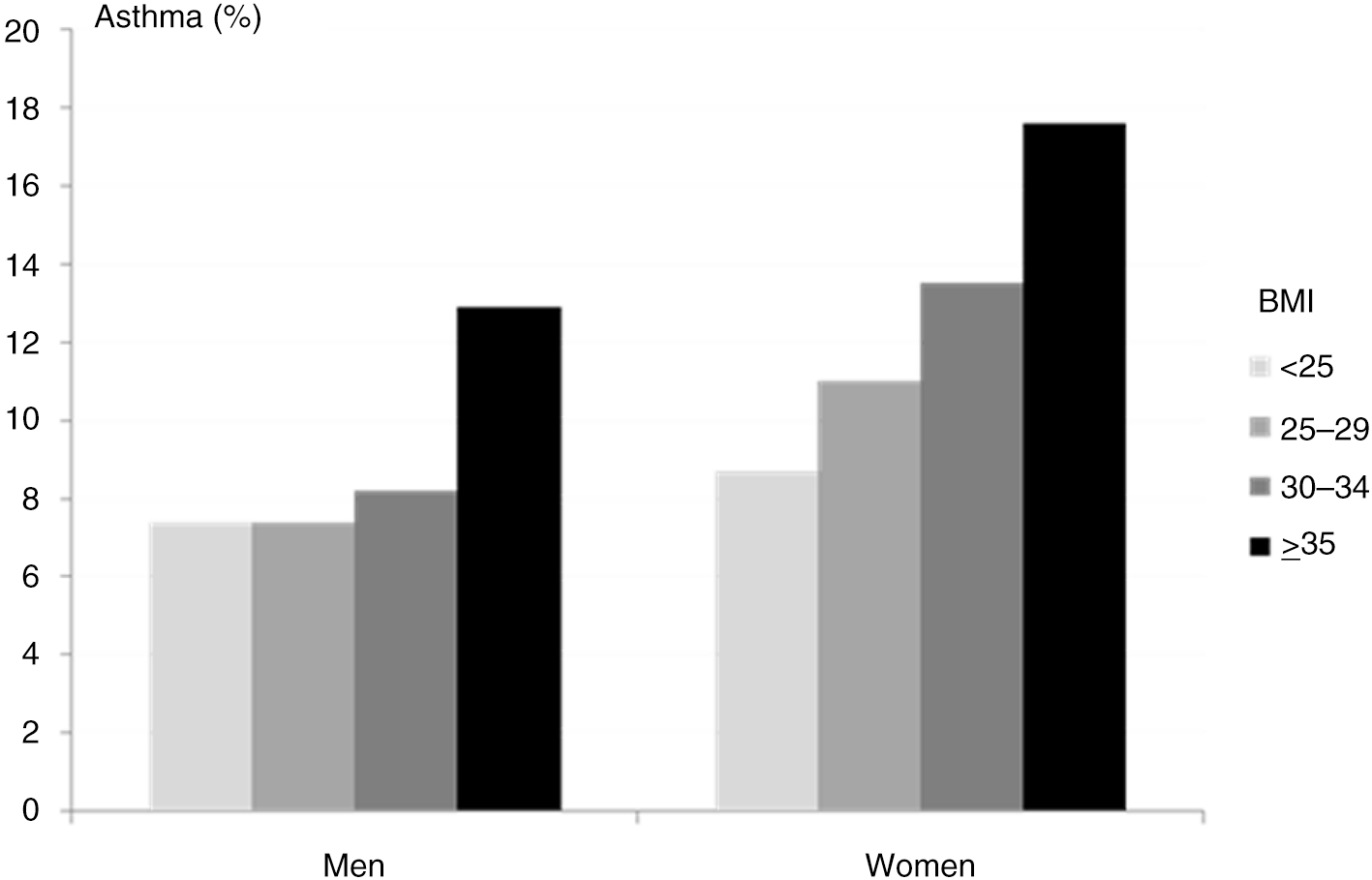
Relationship between asthma and BMI in Danish twins, 20–71 years of age.

Mechanical/physiological factors, systemic inflammation/hormonal factors, and genetics have been proposed to explain the association between obesity and asthma. Particularly, twin studies have suggested that shared genetic pathways for obesity and asthma account, at least in part, for the observed association between these conditions. For example, a Danish study of 34,782 twins, 20–71 years of age showed that BMI was a significant predictor of asthma both in women and in men with a clear dose–response relationship between increasing levels of BMI and asthma (84) (Fig. 3). Furthermore, the risk of asthma was significantly higher in persons with type 2 diabetes compared with those without type 2 diabetes, both in women (16.6% vs. 9.6%) and in men (13.5% vs. 7.5%). Within twin pairs, asthma was more common in the twin with the highest BMI, and with an increased difference in the risk of asthma according to increasing BMI discordance within the twin pair, particularly in DZ twins, consistent with an underlying genetic relationship between obesity and asthma. The genetic correlation between BMI and asthma was estimated to be significant only among women (genetic correlation = 0.15). A significant genetic correlation was also observed between type 2 diabetes and asthma (0.20) (84). A smaller twin study (1,484 pairs) of adults from the United States found a genetic correlation between BMI and asthma of 0.29 (85). A small study of Chinese adults (483 pairs) found that sensitization to common allergens was positively genetically correlated with lipid levels (low levels of HDL and high levels of low-density lipoprotein (LDL)), and percentage body fat, respectively, in men, but not in women (86). However, genetic correlations were not statistically significant except for the association between sensitization and high LDL (genetic correlation = 0.33).

## Conclusions and perspectives

Twin studies have shown that asthma is a highly heritable disease with genetic factors accounting for around 70% of the variation in its susceptibility. In contrast, genetic factors account for only around 35% of the variation in the age at onset of asthma and for around 25% of the variation in the overall symptomatic severity of the disease.

Twin studies have substantiated several leading hypotheses, such as the hygiene hypothesis and the foetal origins hypothesis, as explanations for the modern disease epidemic that apart from asthma also involves autoimmune diseases and the metabolic syndrome. These studies have pointed to a common origin of these chronic inflammatory diseases tracing back to foetal life and early childhood and have revealed that the aetiology of asthma must be understood and further explored in the context of the dynamic cross-talk between genetic factors, (antenatal) developmental factors, and the modern (changing) environment.

Future twin studies of asthma should explore more advanced molecular methods within (epi)genetics (87) and microbiome analysis (88), in order to more specifically understand the aetiology of asthma, and ultimately prevent occurrence and progression of the disease.
